# Prostaglandin E_2_ in a TLR3- and 7/8-agonist-based DC maturation cocktail generates mature, cytokine-producing, migratory DCs but impairs antigen cross-presentation to CD8^+^ T cells

**DOI:** 10.1007/s00262-019-02470-1

**Published:** 2020-02-25

**Authors:** Philipp Gierlich, Veronika Lex, Antje Technau, Anne Keupp, Lorenz Morper, Amelie Glunz, Hanno Sennholz, Johannes Rachor, Sascha Sauer, Ana Marcu, Götz Ulrich Grigoleit, Matthias Wölfl, Paul G. Schlegel, Matthias Eyrich

**Affiliations:** 1 Laboratory for Stem Cell Processing and Cellular TherapyUniversity Medical Center, Children’s Hospital, Würzburg, Germany; 2 CU Systems Medicine, University of Würzburg, Würzburg, Germany; 3 Max Delbrück Center for Molecular Medicine (BIMSB/BIH), Berlin, Germany; 4 Department of Immunology, Interfaculty Institute for Cell Biology, University of Tübingen, Tübingen, Germany; 5 Department of Internal Medicine II, University Medical Center, Würzburg, Germany; 6 University Children’s Hospital Würzburg, Josef-Schneider-Straße 3, Building D30, 97080 Würzburg, Germany

**Keywords:** Dendritic cells, Cancer vaccines, Prostaglandin E_2_, TLR agonists, Tumor-specific CD8^+^ T cells

## Abstract

Mature dendritic cells (DCs) represent cellular adjuvants for optimal antigen presentation in cancer vaccines. Recently, a combination of prostaglandin E_2_ (PGE_2_) with Toll-like receptor agonists (TLR-P) was proposed as a new standard to generate superior cytokine-producing DCs with high migratory capacity. Here, we compare TLR-P DCs with conventional DCs matured only with the proinflammatory cytokines TNFα and IL-1ß (CDCs), focussing on the interaction of resulting DCs with CD8^+^ T-cells. TLR-P matured DCs showed elevated expression of activation markers such as CD80 and CD83 compared to CDCs, together with a significantly higher migration capacity. Secretion of IL-6, IL-8, IL-10, and IL-12 was highest after 16 h in TLR-P DCs, and only TLR-P DCs secreted active IL-12p70. TLR-P DCs as well as CDCs successfully primed multifunctional CD8^+^ T-cells from naïve precursors specific for the peptide antigens Melan-A, NLGN4X, and PTP with comparable priming efficacy and T-cell receptor avidity. CD8^+^ T-cells primed by TLR-P DCs showed significantly elevated expression of the integrin VLA-4 and a trend for higher T-cell numbers after expansion. In contrast, TLR-P DCs displayed a substantially reduced capability to cross-present CMVpp65 protein antigen to pp65-specific T cells, an effect that was dose-dependent on PGE_2_ during DC maturation and reproducible with several responder T-cell lines. In conclusion, TLR-P matured DCs might be optimal presenters of antigens not requiring processing such as short peptides. However, PGE_2_ seems less favorable for maturation of DCs intended to process and cross-present more complex vaccine antigens such as lysates, proteins or long peptides.

**Electronic supplementary material:**

The online version of this article (10.1007/s00262-019-02470-1) contains supplementary material, which is available to authorized users.

## Introduction

Cancer vaccines aim at inducing T-cell responses against particular antigens which segregate tumor cells from non-malignant cells within an organism. Candidate antigens for such a delicate distinction are either derived from non-synonymous mutations in tumor proteins (e.g. neo-antigenic peptides) or tumor-associated antigens (i.e. non-mutated proteins expressed exclusively or aberrantly in tumor cells). Vaccines can contain antigen as tumor lysate, proteins, DNA/RNA, or long peptides. These formats offer the advantage of presentation on both HLA class I and II molecules after intracellular processing to promote simultaneous CD4^+^ and CD8^+^ T-cell responses [1]. Other groups favor mixes of short HLA class I peptides, which bind to membrane-bound HLA class I molecules, do not require processing, and can be synthesized custom tailored for each patient [2]. An inherent problem with cancer antigens is that TCRs with a specificity for tumor-associated peptides have at least one log lower affinity for their target peptide-MHC complex than TCRs with viral specificity [3]. To compensate for this and yet allow appropriate sensing of the applied antigen by the immune system, an optimal presentation of cancer vaccine antigens is mandatory. Chemical adjuvants commonly used in vaccination trials such as alum salts, Montanide, or GM-CSF suffer from substantial shortcomings such as promoting Th2 responses, inducing dysfunctional T-cells or myeloid-derived suppressor cells [2, 4, 5], making the search for better adjuvants a major goal in cancer vaccine development.

Naturally occurring professional antigen-presenting cells such as DCs potentially might overcome many of the drawbacks of synthetic adjuvants. DCs actively exert important vaccine promoting functions in vivo, such as antigen transport via targeted migration into secondary lymphoid organs, longevity, and phenotype stability for at least 3–4 days, cross-presentation of ingested antigen to CD8^+^ T-cells, and secretion of appropriate cytokines at the DC:T synapse, ultimately leading to successful priming of naïve T-cells and memory formation. However, as DCs are a highly versatile cell population, there is no consensus on how to prepare DC precursors for their role in a cancer vaccine. Historically, conventional dendritic cells (CDCs) have been generated from monocyte precursors by inducing differentiation towards an immature DC state with IL-4 and GM-CSF and after antigen loading in the phagocytic stage, maturation with a cytokine-based cocktail containing TNFα, IL-1ß, IL-6 and prostaglandin E_2_ (PGE_2_) [6]. However, evidence emerged that some essential functions (e.g. migratory and cytokine secretion capabilities) might be mutually exclusive in monocyte-derived DCs and that PGE_2_ is a decisive switch in that process [7]. Production of IL-12p70, an important cytokine influencing the properties of CD8^+^ T- and NK-cells, was found to be impaired by PGE_2_ [8] and presence of PGE_2_ during iDC maturation resulted in an IL-10 dependent abrogation of antigen-specific T-cell proliferation [9]. Furthermore, PGE_2_ can trigger a COX-2-driven positive feedback loop in monocytes and redirect their differentiation into MDSCs [10]. Altogether, these data led to removal of PGE_2_ from DC maturation cocktails by several groups [11]. In our own brain tumor vaccination program, we have used TNFα and IL-1ß as maturing agents so far and validated this combination as the standard for generation of clinical-grade DCs under GMP [12, 13].

TLRs are innate receptors on antigen-presenting cells sensing pathogen-associated molecular patterns in antimicrobial defense. Their triggering induces potent activation of DCs, harnessing them with competence for optimal antigen transport and T-cell activation. Combinatorial triggering of the TLR3/4 and TLR7/8/9 subgroups was shown to synergistically activate a type-1 polarizing program for T helper cells together with high IL-12/-23 production and a specific transcriptome in DCs [14] through MyD88-dependent and -independent pathways [15]. TLR-matured DCs exclusively activated CD8^+^ T-cells with granzyme B/chemokine receptor (CCR-)5 expression and CTL activity [16]. These findings were rapidly translated into DC manufacturing protocols in the cancer vaccine setting. PolyI:C, a TLR3 agonist which mimics viral double stranded RNA (dsRNA), was added to a cytokine-based DC maturation cocktail, resulting in increased IL-12p70 production by DCs and a superior induction of melanoma-specific CTL responses [17]. Enhanced stimulation of tumor-specific T-cells was achieved when polyI:C and R848 (Resiquimod, a synthetic low-molecular-weight agonist of TLR7/8) were combined with LPS, the classical TLR4-ligand [18]. Furthermore, researchers were interested to analyze whether the combined use of TLR-agonists and PGE_2_ was able to generate DCs with both cytokine-secreting and migratory capabilities. Boullard et al. investigated the effect of adding PGE_2_ to polyI:C and R848 on DC phenotype, migratory capacity, IL-12p70 production, and T-cell cytokine secretion. They concluded that the combination of TLR3&7/8 agonists with PGE_2_ (TLR-P) results in DCs with improved migratory capacity and maintained IL-12p70 production [19]. However, important DC functions such as DC:T interactions were not assessed in that report. Here, we compare our extensively validated TNFα/IL-1ß cytokine combination, already used in clinical trials, with the proposed polyI:C/R848/PGE_2_ cocktail with a special focus on the interaction of resulting DCs with CD8^+^ T-cells. We confirm previous data that TLR-P matured DCs exhibit a mature phenotype and robust migratory and cytokine-secreting abilities. Furthermore, CDCs and TLR-P DCs are equally able to successfully prime antigen-specific CD8^+^ T-cell responses against tumor-associated antigens. However, we show for the first time that PGE_2_ might impair cross-presentation of protein antigens to CD8^+^ T cells in human DCs in a dose-dependent fashion. Our data have implications for choosing the optimal DC maturation cocktail, which should be adapted to the type of antigen used in a cancer vaccine.

## Material and methods

### DC generation

PBMCs were obtained from anonymized, surplus leukocyte reduction filters from thrombocyte donations. DCs were generated as previously described [12]: monocytes were purified by CD14-MACS as recommended by the manufacturer (Miltenyi Biotec, Germany). Sorted cells were resuspended in CellGro DC medium (CellGenix, Freiburg, Germany) containing 1vol% HSA at a concentration of 1.5 × 10^6^ cells/ml and plated on tissue culture flask (Nunclon™ Delta, Thermo Fisher Scientific). For differentiation of monocytes into immature DCs (iDCs) IL-4 (1000 U/ml) and GM-CSF (1000 U/ml) were used for 5 days (both from CellGenix). Fresh medium and cytokines were added on days 2 and 4. On day 5, DC maturation was induced by addition of either TNFα (1000 U/ml) and IL-1ß (2000 U/ml) (cocktail 1) or R848 (3 μg/ml, Invivogen), Poly I:C (20 μg/ml. Sigma-Aldrich), and PGE_2_ (10 µg/ml, Sigma-Aldrich) (cocktail 2). The main objective of this study was to compare the impact of these two protocols on DC maturation. In some selected experiments, PGE_2_ was omitted or dose-modified to highlight the role of this substance in the TLR-cocktail. After 16–48 h maturation, viable mature DCs (mDCs) were harvested by putting on ice for 20 min, repeatedly rinsed with cold PBS) pooled if DCs from the same donor and condition were cultured in separate wells, counted, and analyzed for further assays (see below). General yield of mature DCs from the starting monocyte population in this assay was 51.4% (35.2–80.6) (median and range, data not shown). DCs for experiments on phenotype, migration, and cytokine secretion were matured for 48 h as in the index publication [19]. For T-cell experiments, a 16 h DC maturation period was strongly recommended in the original publications [20, 21], and backed by our own data. Thus, for T-cell priming and cross-presentation experiments, a 16 h maturation period was chosen. Furthermore, to investigate the longevity and phenotype stability of matured DCs, we extended the culture period in some experiments for another 6 days. During this extended culture, DCs were washed and received only low-dose IL-4 and GM-CSF (250 U/ml) to avoid DC starvation but no further maturing agents.

For cross-presentation experiments, CD141^+^ DCs served as positive controls. To enrich this very low frequency DC subpopulation, PBMCs from HLA-A02*01 donors were incubated with blood-derived dendritic cell antigen 3 (BDCA-3) microbeads (Miltenyi Biotec) and positively enriched on LS-MACS columns according to the manufacturer’s instructions. Due to the scarcity of events, the positive fraction was only enumerated in Neubauer chambers.

### Flow cytometry

Monocytes, iDCs (day 5), and mDCs (day 7) were stained for expression of CD14 (clone MϕP9, APC), C86 (clone 2331 (FUN-1), FITC), CD80 (clone L307.4, PE), CD83 (clone HB15e, PE), HLA-DR (clone G46-6, APC), and DC-SIGN (clone DCN46, APC) in some experiments. Dead and pre-apoptotic cells were excluded by addition of 7-aminoactinomycin (7-AAD). Since CD86 and HLA-DR are constitutively expressed on most monocytes and DCs, the increase in MFI of these markers was used to assess maturation. For CD80 and CD83, the increase in frequency of positive cells was noted. Cytometer settings were optimized for mDCs, and the same settings were used on day 0 and 7 (with an threshold adjustment for the higher auto-fluorescence on day 7) so that MFI-values could be compared. T cells were stained for CD3 (clone HIT3a, PE), CD4 (clone SK3, PerCp), CD8 (clone SK1, PerCp), CD62L (clone DREG-56, APC), CD45RA (cloneHI100, FITC), CD45RO (clone UCHL1, PE), CD137 (clone 4B4-1, APC), VLA-4 (CD29 (clone TS2/16, FITC) / CD49d (clone 9F10, PE) heterodimer), IFNγ (clone B27, PE), TNFα (clone 64.011.111, APC), and the indicated MHC-multimers. All antibodies were obtained from BD (BD Biosciences, Heidelberg, Germany). Customized MHC-multimers (APC) were purchased from Proimmune (Oxford, UK). Cells were stained using standard four-color or intracellular (BD Fix&Perm) procedures and a minimum of 10,000 events (for MHC-multimer and IFNγ-analysis 100.000 events) were acquired on a FACSCalibur (BD). Quality assurance of the flow cytometer included weekly calibration and internal quality controls as well as four monthly external round robin tests. Isotype controls for all colors were included in all measurements. Results were analyzed with CellQuest Pro (version 5.2.1, BD) or FlowJo (version 9.6, TreeStar, Ashland, Oregon, USA).

### Migration assay

Migration assays were performed in 24-well Transwell plates carrying permeable polycarbonate filters of 5 µm pore size (Corning Life Sciences). mDC were washed thoroughly and suspended in CellGro media at 10^6^/ml. The lower chambers of the Transwell were filled with 600 µl of CellGro containing 25 ng/ml of chemokine ligand (CCL-)19 and CCL21 each (both from PeproTech). 100 µl of the mDC were added into the upper chamber and allowed to migrate for 3 h in a 5% CO_2_, humidified incubator at 37 °C. 500 µl aliquot of the cells that had migrated to the lower chamber was counted by flow cytometry for a fixed period of 300 s at a constant speed. Values are given as median counts of migrated cell with range.

### Cytokine secretion

Cell culture supernatant (200 µl) was collected after centrifugation from each well after 8, 16, and 48 h of maturation and stored at − 20 °C until analysis. Analytes were measured according to the manufacturers instructions on a MagPix device (Luminex, Oosterhout, The Netherlands) using the Human Magnetic Multiplex Kit (LHC6003M, Lifetechnologies, Darmstadt, Germany). The Luminex assay includes an internal calibration set, high and low validation samples and a 7-point curve for standard generation. Analytes not fulfilling these internal validations or showing no elevation above the baseline (iDCs before maturation) were not included into the analysis. IL-12p70 was measured additionally in a 96-well ELISA (ThermoFischer, Langenselbold, Germany). Samples were prepared according to the manufacturer’s instructions and measured at 450 nm on a Genios plate reader and analyzed using Magellan Software (Tecan, Crailsheim, Germany).

### T-cell assays

For T-cell priming assays, we used a previously described protocol [20, 21]. In brief, naive CD8^+^ T cells were isolated from the peripheral blood of healthy donors using a purification of CD8^+^ events, followed by depletion with CD45RO and CD57 microbeads on LD and LS columns (Miltenyi, Bergisch Gladbach, Germany). Isolated naive CD8^+^ T cells were incubated with IL-7 overnight. The next day, T cells were washed and mixed with irradiated, differently matured autologous DCs, pulsed with either the heteroclitic HLA-A02*01-restricted peptide Melan-A_26-35L_ as a control or alternative peptides as indicated at 1 µg/ml. IL-21 (30 ng/ml, Peprotech) was added to the co-culture on day 0 the next morning. IL-7 and IL-15 (5 ng/ml, Peprotech) were added on days 3, 5, and 7 and cells were harvested on day 10, some of them after restimulation with the original peptide. The Melan-A_26-35L_ (ELAGIGIGLTV, NetMHC 4.0 affinity 1738.18 nM), neuroligin-4, X-linked (NLGN4X) (NLDTLMTYV, affinity 8.89 nM), and tyrosine-protein phosphatase non-receptor (PTP) (KVFAGIPTV, affinity 5.87 nM) peptides were purchased from jpt (Berlin, Germany), the CMV-peptide NLVPMVATV was synthesized by the peptide facility of the Department of Immunology, University of Tübingen. Harvested T-cells on day 10 or 12 were analyzed for phenotype (CD3, CD8, CD62L, CD45RA, VLA4), MHC-multimer- and TNFα/IFNγ-positivity. TCR avidity was determined by stimulating harvested day10-T-cells with autologous monocytes loaded with peptide concentrations ranging from 1 µg/ml to 1 pg/ml at a 4:1 ratio for 5 h and assaying for TNFα and IFNγ-production.

Two independent responder T-cell lines with specificity for the HLA-A02*01-restrcted CMV-peptide NLVPMVATV were established by stimulating PBMCs from a CMV-seropositive healthy donor with 1 µg/ml peptide and stimulating αCD28-mAb at 1 µl/ml (Miltenyi Biotec, Bergisch-Gladbach, Germany). After 24 h stimulation, CD137^+^ cells were positively isolated by magnetic CD137-cell sorting on MACS columns (Miltenyi Biotec) [22]. The positive fraction was expanded for 11–15 days with IL-21 (10 ng/ml), IL-7, and IL-15 (each 5 ng/ml) in CellGro Medium (CellGenix, Freiburg, Germany) supplemented with 1% P/S, and 5% human serum. New medium and cytokines were supplemented every 2–3 days. After 9 days. T-cells were restimulated with peptide-pulsed, irradiated allogeneic feeder cells and aCD28 costimulatory mAbs. Specificity of the cell lines was measured by binding of the HLA-A02, CMV MHC-multimer (APC, Immundex, Copenhagen, Denmark) on CD8^+^ T cells. Target value was more than 70% MHC-multimer-positive T-cells after the end of culture. T-cells were used either freshly or were cryopreserved for later use.

### Cross-presentation assays

For cross-presentation experiments, different DC subtypes were differentiated with IL-4 and GM-CSF as indicated above. Freshly isolated CD141^+^ (BDCA-3) DCs served as a positive control. On day 6, in half of the wells CMVpp65 recombinant protein (10 µl/ml, Miltenyi Biotec) was added and DCs were subsequently matured for another 16 h as indicated above. In some experiments, PGE_2_ was omitted from or titrated into the TLR-agonist cocktail. BDCA-3 DCs were matured with polyI:C (20 ng/ml). Negative controls contained unloaded DCs, T-cells without stimulation and in some experiments (when sufficient CD141^+^ DCs were available) also CD8^+^ T cells from a CMV-seronegative donor against protein-loaded CD141^+^ DCs. After maturation, the other half of the wells were pulsed with the NLVPMVATV peptide (1 µg/ml) for 1 h. Afterwards, all DCs were washed, resuspended at a concentration of 5 × 10^5^/ml and 100 µl plated in a 96-well. 100 µl of the CMV-responder cell line (2 × 10^6^/ml) were added in the presence of Brefeldin (Sigma, Taufkirchen, Germany) and co-incubated for 7 h at 37 °C, 5% CO_2_ (DC:T ratio 1:4). Subsequently, cells were permeabilized and stained for IFNγ/TNFα as outlined above. Results were calculated as relative deviation from the positive control (CD141^+^ DCs = 100%) in the respective experiments.

### Statistical analysis

Intergroup differences were assessed by nonparametric Mann–Whitney *U* test assuming unequal variances between groups. For comparison of multiple groups, the non-parametric Kruskal–Wallis rang-sum test was used. For differences within a group the two-tailed *t *test was applied. *p* < 0.05 was considered statistically significant.

## Results

### Effect of cytokine versus TLR-agonist-based cocktails on DC maturation marker expression

**Fig. 1:**
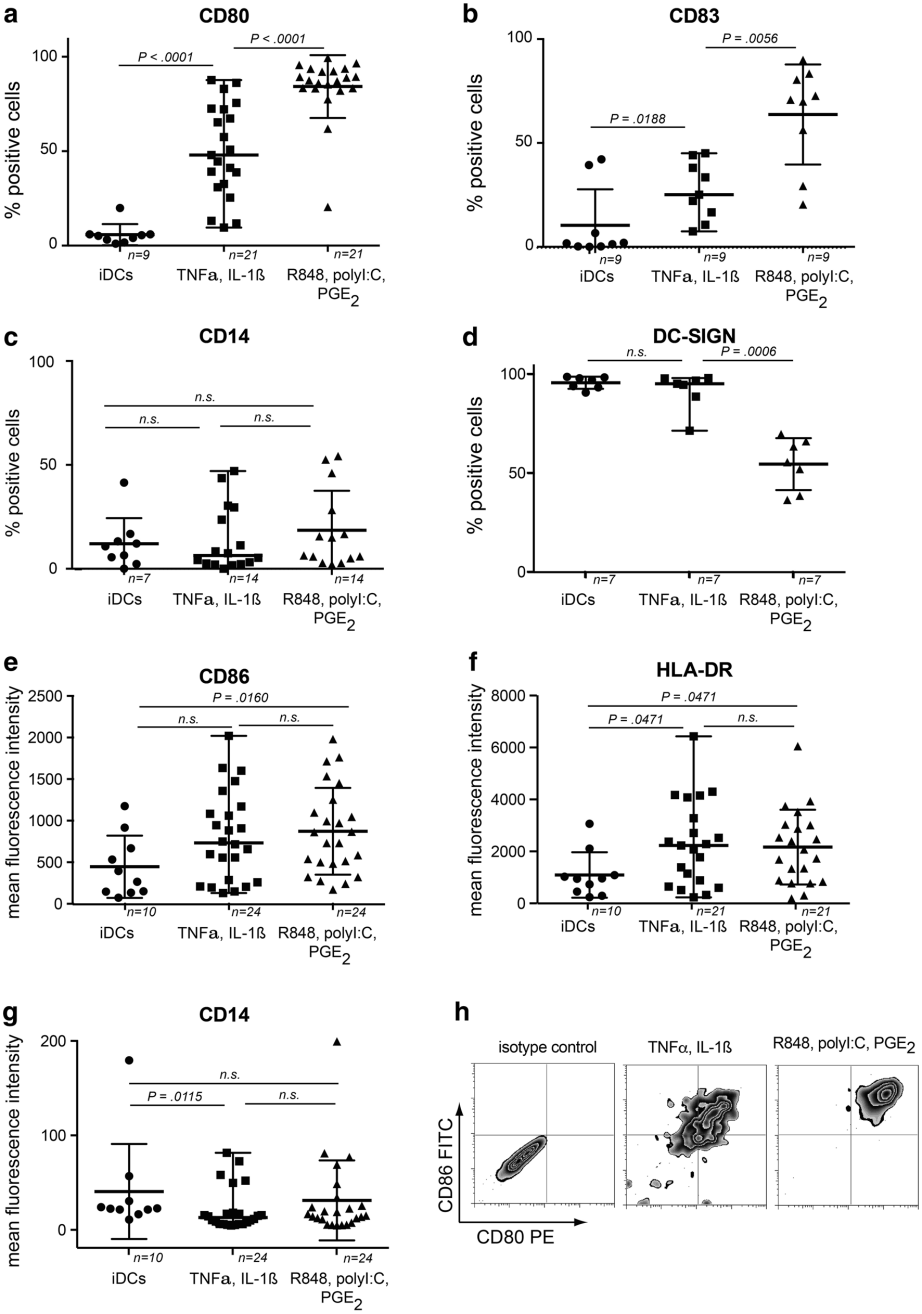
Expression of DC surface maturation markers. DCs were either analyzed in an immature state on day 7 or after 48 h maturation. For CD80 (**a**), CD83 (**b**), CD14 (**c**), and DC-SIGN (**d)** percentages of marker positive cells were determined, for the constitutive markers CD86 (**e**), HLA-DR (**f**), and CD14 (**g**) the change in fluorescence intensity is displayed. Gating strategy is detailed in supplementary Fig. 1a. **h** Representative examples of day7-mDCs. *N* indicates the number of experiments, *n.s.* not significant, line and error bars represent medians and ranges

First, we analyzed the differential effects of the cytokine-based (TNFα/IL-1ß) *versus* TLR-P cocktail (R848/polyI:C/PGE_2_) on DC maturation marker expression. Both cocktails resulted in a significant upregulation of CD80, CD83, CD86, and HLA-DR on day7-mDCs compared to day7-iDCs (Fig. 1). However, the TLR-P-cocktail resulted in significantly more CD80^+^ and CD83^+^ DCs (Fig. 1a+b) as well as higher expression of CD86 and HLA-DR (Fig. 1e+f). CD14 expression was lower on cytokine matured than on iDCs or TLR-P-mDCs (Fig. 1g), but percentages of residual CD14^+^ cells after maturation were not different between the three groups (Fig. 1c). In contrast, DC-SIGN (CD209) which was already high on day7-iDCs significantly declined on TLR-P-matured DCs, but not on CDCs (Fig. 1d).

### mDC life span, phenotype stability, and migration capacity

**Fig. 2:**
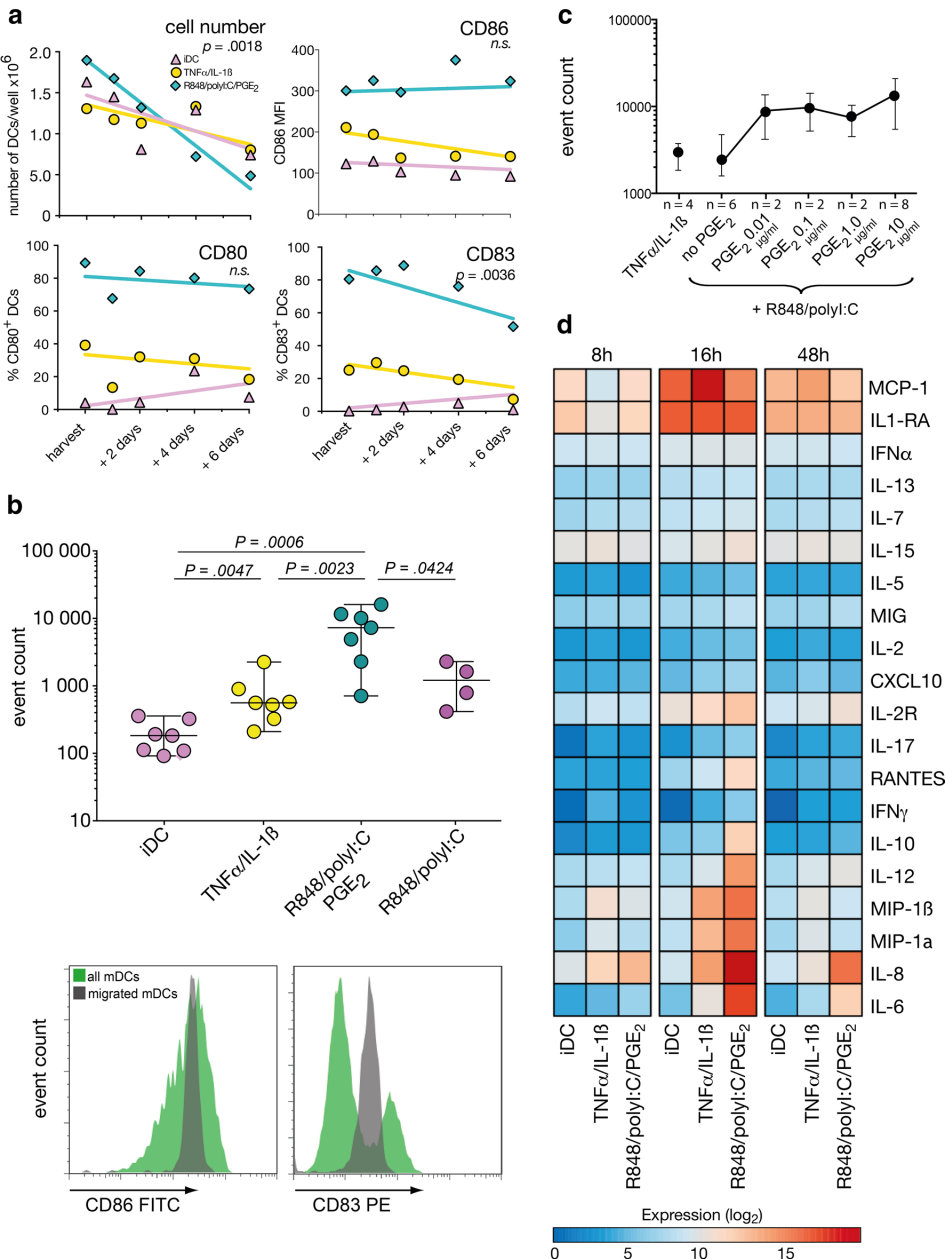
Stability, migration, and cytokine secretion of differentially matured DCs. **a** After harvesting on day +7, DCs were maintained, counted, and phenotyped for another 6 days. Gating strategy is detailed in supplementary Fig. 1a. Each symbol represents the median of *n* = 5 experiments, differences of slopes were calculated by linear regression. **b** Number of DCs migrating along a CCL19/21 gradient in a transwell assay. Gating strategy for identifying DCs is detailed in supplementary Fig. 1b. Line and error bars represent medians and ranges, each symbol represents one experiment. Histograms show representative examples of CD86 and CD83 expression on all *vs.* migrated DCs. **c** DCs were matured with titrating doses of PGE_2_ and assayed for targeted migration as described above, medians with ranges. **d** Heat map of cytokine concentrations in supernatants after 8, 16 and 48 h of maturation. Data from *n* = 7, *n* = 3 and *n* = 10 experiments, respectively were log_2_-transformed

Next, we were interested to see whether the two maturation cocktails had different long-term effects on DCs in terms of cell number and phenotype stability. To analyze this we washed out all maturing agents on day 7 and extended DC culture for another 6 days in medium supplemented only with low dose IL-4 and GM-CSF with the intention to avoid DC starvation. Under these conditions, cell numbers gradually declined, after TLR-P maturation significantly faster than in cytokine-matured or immature DCs (Fig. 2a). CD86 and CD80 expression remained fairly constant, whereas CD83 expression declined over time in both matured *versus* immature DCs (Fig. 2a).

Directed migration towards secondary lymphatic organs along a lymphokine gradient represents one of the first actions a DC has to complete after injection. Using a CCL19/21-dependent migration assay, we could show that TNFα/IL-1ß induced significantly enhanced migration compared to iDCs, however, the TLR-P cocktail further boosted migration by more than one log. When PGE_2_ was omitted in the TLR-agonist cocktail, the number of actively migrating cells significantly diminished, indicating that migratory capabilities in DCs optimally develop in the presence of PGE_2_ (Fig. 2b). Titration of PGE_2_ in the maturation cocktail from 10 ng/ml to 10 µg/ml showed that already low PGE_2_ doses of 10 ng/ml resulted in increases of migratory capacity whereas further augmentation of the PGE_2_ dose had only moderate effects (Fig. 2c). Migrated DCs from the lower chamber displayed a more uniform activation marker expression, suggesting that only a subfraction of mature DCs is endowed with the capacity to migrate or that migration impacted expression levels (Fig. 2b, lower histograms).

### DC cytokine secretion

Cytokine secretion of DCs followed a clear time-dependent pattern: while only few cytokines were produced 8 h after start of maturation, secretion generally peaked after 16 h, and then slowly declined afterwards (Fig. 2d). With the exception of MCP-1, TLR-P-matured DCs showed the highest cytokine release which was significant at 16 h for IL-6, IL-8, IL-10, and IL-12 (Table 1). The IL-12/IL-10 ratio was positive at 16 and 48 h (Table 1), the increase at 48 h was caused by a faster drop of IL-10 compared to IL-12 values.

**Table 1:** Cytokine secretion by DCs after maturation, median [ranges]

	Unit	TLR-P DCs	CDCs	
*Luminex assay after 16 h maturation*				
IL-6	[pg/ml]	97,855 [57,990–98,716]	1395 [124–2881]	*p* = 0.0001
IL-8	[pg/ml]	384,553 [369,029–588,577]	20,977 [1930–42,566]	*p* = 0.0008
IL-10	[pg/ml]	1669 [1454–3038]	71 [35–109]	*p* = 0.053
IL-12_p35/p40_	[pg/ml]	10,237 [9707–11,416]	310 [166–933]	*p* = 0.004
IL-12_p35/p40_/IL-10 after 16 h	Ratio	6.7 [3.4–6.8]	5.0 [4.7–8.6]	n.s
IL-12_p35/p40_/IL-10 after 48 h	Ratio	58 [16–1731]	27 [6–1919]	n.s
*ELISA assay*				
IL-12_p70_ after 8 h	[pg/ml]	14.7 [13.3–16.2]	0	
IL-12_p70_ after 16 h	[pg/ml]	75.1 [70.1–80.1]	0	
IL-12_p70_ after 48 h	[pg/ml]	51.2 [26.7–75.7]	0	

Since the Luminex assay measures IL-12_p35/p40_, we additionally selectively assayed the biologically active IL-12_p70_ by ELISA: IL-12_p70_ was only detectable in TLR-P-matured DCs, whereas no IL-12_p70_ could be detected in supernatants of iDCs or CDCs at any time (Table 1). Since IL-12_p70_ and IL-10 were measured in two different assay systems, we did not determine the IL-12_p70_/IL-10 ratio.

### De novo priming of tumor-antigen-specific T-cells

**Fig. 3:**
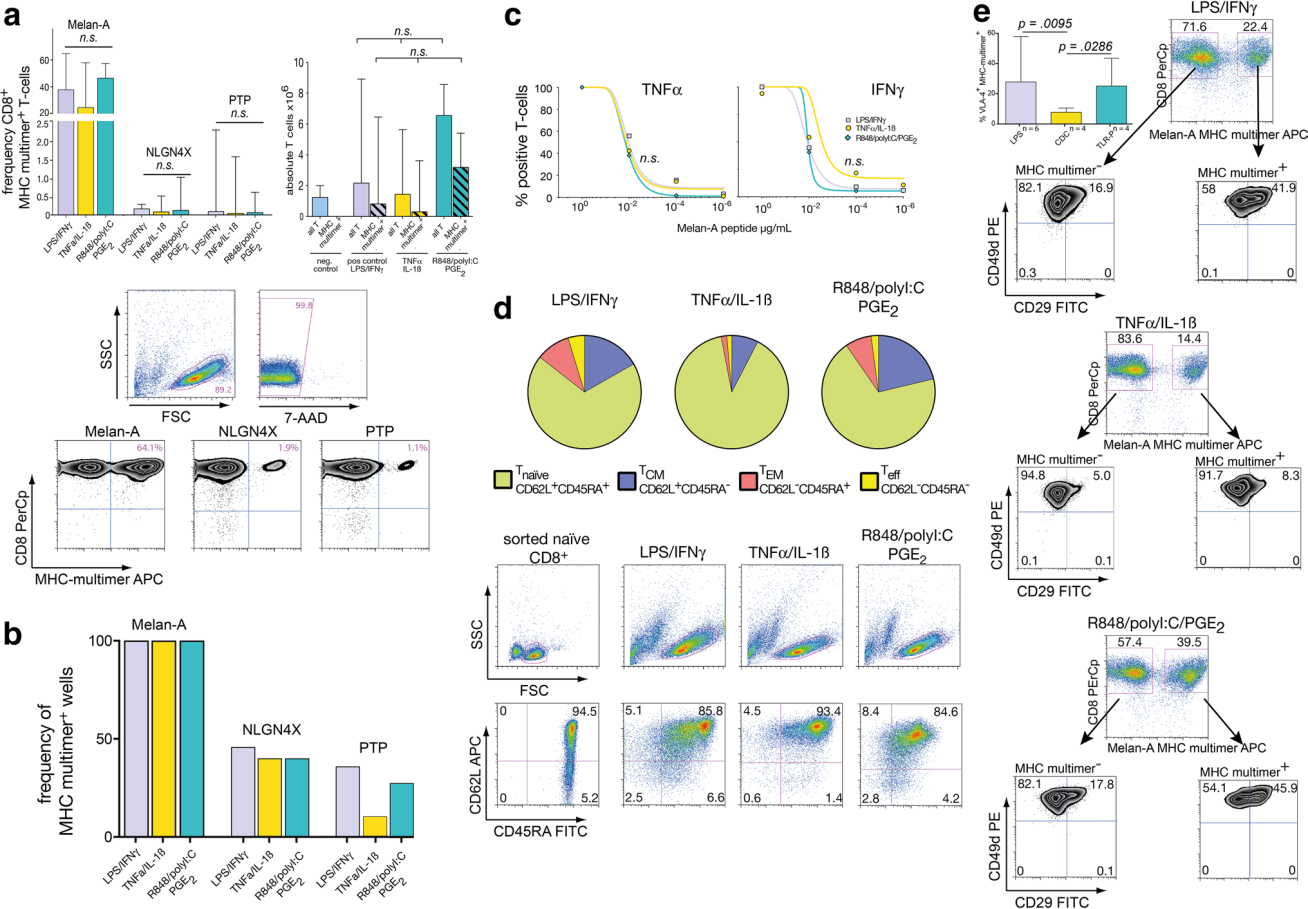
T-cell priming capacity of differentially matured DCs. **a** Naïve CD8^+^ T-cells were primed against the peptides Melan-A_26-35L_, NLGN4X_131-139_, and PTP_1347-1355_ using differentially matured DCs. Medians and ranges from *n* = 3 experiments are shown. Dot plots depict gating strategy and representative MHC-multimer stainings. **b** From the same experiments the percentage of wells assessed as multimer^pos^ (threshold > 0.1%) was calculated. **c** TCR avidity was determined by restimulating T-cells with titrating dosages of peptide. Values at 1 µg/ml were set as 100% and all other values normalized to that maximum. Medians of *n* = 4 experiments, gating strategy is detailed in supplementary Fig. 1c. **d** Harvested day10-CD8^+^-T-cells were phenotyped and categorized into T_naive_, T_CM_, T_EM_, and T_eff_ subsets. Pie charts show median values of *n* = 3 experiments, dot plots display gating strategy and representative examples. **e** Analysis of integrin VLA-4 expression on harvested day10-CD8^+^ T-cells depending on MHC-multimer positivity. CD8^+^ T cells were identified as shown in supplementary Fig. 1c. Columns represent median values with ranges, n indicates the number of experiments

Many cancer patients do not harbor pre-existing T-cell responses against tumor-associated antigens. Thus, de novo T-cell priming is of pivotal importance for functionality of cancer vaccines. To test the differential ability of our DCs to prime T-cells, we established an in vitro priming assay, in which highly purified naïve CD8^+^ T-cells are primed against peptide-loaded autologous DCs and subsequently expanded. As described in the original publication, LPS/IFNγ-matured DCs served as a positive control and all assays included the heteroclitic Melan-A_26-35L_ peptide, which usually gives high-frequency responses [20]. To represent also antigens with lower precursor frequencies, we chose the previously described, glioma-associated NLGN4X_131-139_ and PTP_1347-1355_ peptides [23]. As shown in Fig. 3a (left graph), all three types of DCs induced high-frequency Melan-A_26-35L_ responses, whereas the two glioma peptides resulted in substantially lower, but detectable CD8^+^ T-cell responses. No significant differences in terms of primed MHC-multimer^+^ T-cells between differently matured DC populations could be detected. Likewise, TLR-P DCs tended to induce greater proliferation of T cells compared to CDCs (Fig. 3a right graph). In line with the known high precursor frequency of Melan-A_26-35L_ reactive T-cells in the naïve compartment (approximately 0.1%, i.e. 1/10^3^), all wells contained Melan-A_26-35L_ specific T-cells, whereas for NLGN4X_131-139_ and PTP_1347-1355_ only a fraction of 10–46% of wells contained positive cells (Fig. 3b), resulting in a minimal naïve precursor T-cell frequency of at least 1/1.2 × 10^6^ for NLGN4X_131-139_ and 1/2.04 × 10^6^ for PTP_1347-1355_. Due to limiting cells numbers, TCR avidity of induced T-cells could only be assayed for Melan-A-specific T-cells. To test this, harvested T-cells from day 10 were restimulated with autologous monocytes pulsed with titrated peptide dosages from µg- to pg-ranges. We found no difference in the TNFα- or IFNγ-cytokine responsiveness of T-cells primed by differently matured DC subgroups (Fig. 3c). The phenotype of the whole expanded T-cell pool on day 10 showed a trend for more effector T-cells with the TLR-matured DCs: starting from a homogenous naïve T-cell population, CD62L and CD45RA were subsequently downregulated and most T-cells were in naïve-memory transition after 10 days of expansion under these conditions (Fig. 3d). Gating on the primed MHC-multimer^+^ T-cells, we found that the expression of the CD49d/CD29 heterodimer (VLA-4), which represents a key homing receptor for CNS tissues significantly differed between T-cells induced by differently matured DCs. VLA-4 expression was more abundant on MHC-multimer^+^ than on MHC-multimer^−^ T-cells and was higher after T-cell stimulation with TLR-matured DCs (Fig. 3e). In conclusion, all three DC populations were able to prime antigen-specific CD8^+^ T-cells from antigens with low and intermediate precursor frequency. We were not able to detect significant differences regarding frequency and TCR avidity of T-cells resulting from priming with the three DC subpopulations, however, on the phenotype level, TLR-matured DCs tended to induce more effector T-cells with higher VLA-4 expression.

### Cross-presenting capacity

**Fig. 4:**
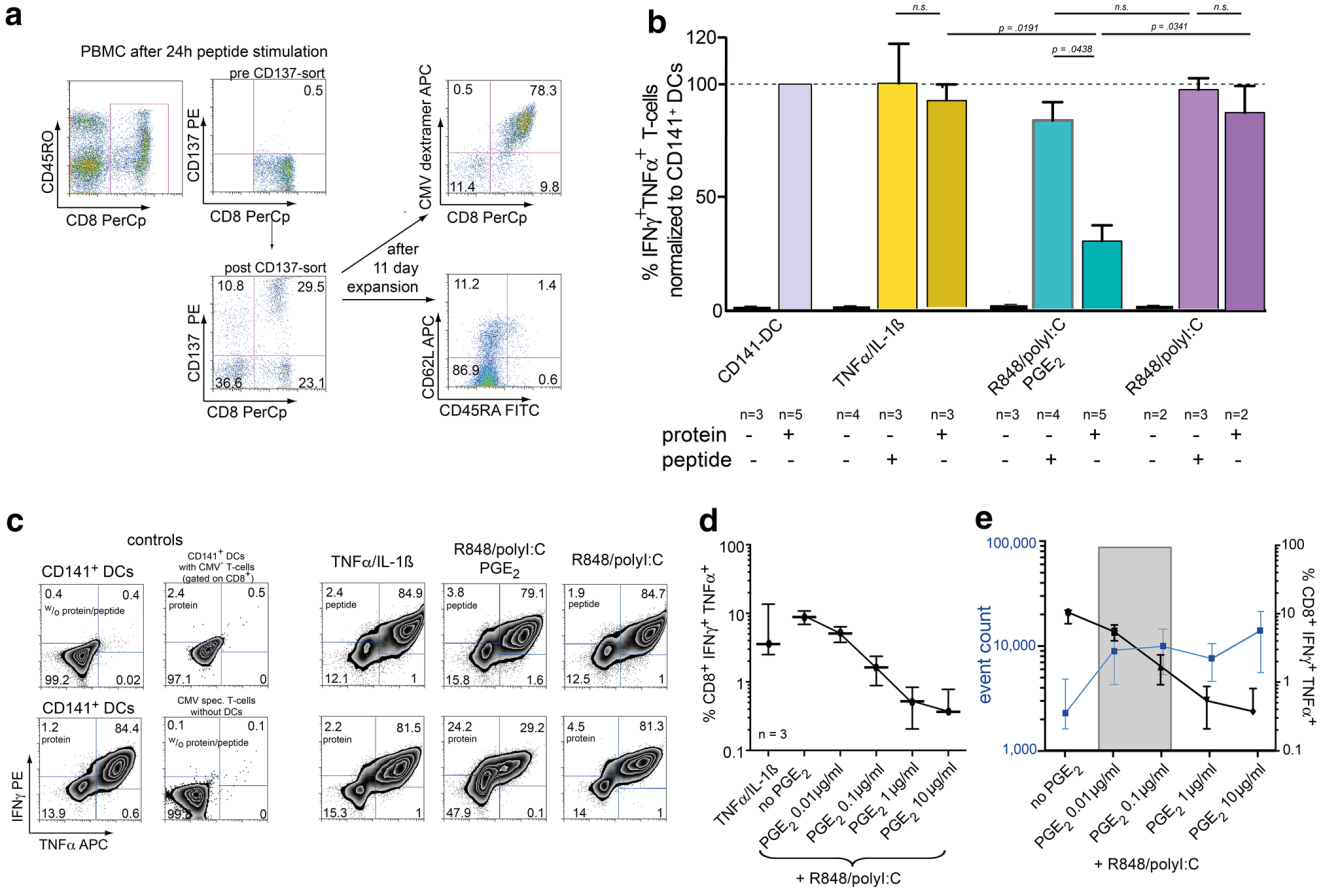
Cross-presenting capabilities of differently matured DC subsets. **a** Representative FACS plots of key steps in the generation of CMV responder cell lines. **b** Frequency of cytokine producing, CMV-specific responder CD8^+^ T cells after stimulation with different DC subtypes and antigen sources. Results were normalized to the positive control (BDCA-3 DCs). Columns represent medians with ranges, n the number of experiments, n.s. non-significant. **c** Representative examples of polyfunctional T-cells (gated on SSC^low^CD8^+^CMV-MHC-multimer^+^). **d** Frequency of cytokine producing, CMV-specific responder CD8^+^ T cells after stimulation with DCs matured under titrated PGE_2_ dosages, lines and error bars indicate medians and ranges, *n* the number of experiments. **e** Overlay of Fig. 2c and 4d. Gray area highlights a potentially preferable PGE_2_ concentration range

Finally, we wanted to investigate whether the different DC maturation cocktails affect the ability of DCs to cross-present phagocytosed material to CD8^+^ T-cells. To this end, we incubated iDCs in the phagocytic stage with the full length CMVpp65 protein, and matured them afterwards using the different cocktails. The ability of these differentially matured DCs to stimulate CMVpp65-specific polyfunctional (TNFα^+^IFNγ^+^) CD8^+^ T-cell responses was tested by co-incubating them with three different responder T-cell populations: either two CMVpp65-specific T-cell lines generated in our own lab or a CMVpp65 clone (kindly provided by F. Falkenburg and I. Jedema, Leiden). HLA-A*02^+^ donors with a clearly detectable CD8^+^CMV-MHC-multimer^+^ population were used to generate two T-cell lines with a final CMV specificity of > 75% (Fig. 4a). CD141^+^ (BDCA-3) DCs isolated from peripheral blood of HLA-A*02:01 donors were chosen as a positive control, as these DCs are considered to be the human APC population with most potent cross-presenting and T-cell stimulating function [24]. Further positive controls included peptide loaded DCs and PMA/Ionomycin stimulated T-cells (not shown), negative controls unloaded DCs, unstimulated T cells as well as CD8^+^ responder T-cells from CMV-seronegative donors (Fig. 4c). Unloaded DCs did not stimulate CMV-specific responses, ruling out endogenous expression of CMVpp65 antigen by our DCs. As illustrated in Fig. 4b+c, CD141^+^ DCs most effectively cross-presented the CMVpp65 protein, yielding the highest frequency of CD8^+^IFNγ^+^TNFα^+^ T-cells (these values were subsequently defined as 100%). As expected, peptide-loaded DCs tended to result in slightly higher cytokine production, cross-presenting CDCs also gave non-inferior results. In contrast, TLR-P DCs showed significantly lower frequencies of CD8^+^IFNγ^+^TNFα^+^ T-cells compared both to their CDC counterparts as well as their internal peptide controls (Fig. 4b+c). This effect was reproducible in all three T-cell responder populations. To pinpoint the effect of PGE_2_ in that assay, we excluded PGE_2_ from the TLR-P-cocktail. Omission of PGE_2_ restored cross-presenting function of TLR-matured DCs back to normal levels. Furthermore, titration of PGE_2_ in the maturation cocktail from 10 ng/ml to 10 µg/ml demonstrated that this effect of PGE_2_ was dose-dependent (Fig. 4d). These data indicate that PGE_2_ specifically inhibits cross-presentation of protein antigen to CD8^+^ T-cells in human TLR-matured DCs.

Since cross-presenting and migratory capacities seem to be inversely regulated by PGE_2_, we superimposed these data. The resulting overlay graph (Fig. 4e) suggests that concentrations of PGE_2_ in the range of 10–100 ng/ml might be an optimal dose window where migratory capacity is already upregulated yet cross-presenting function is not totally abrogated.

## Discussion

Manufacturing of DCs under GMP conditions for use as a clinical-grade cancer vaccine aims at generating a homogenous cell population fulfilling predefined specifications with a Th1-promoting profile. In vitro conditions shield the cells from potentially harmful factors in a cancer-bearing host, and the manufacturing process should be adapted to the type of antigen used in the vaccine, e.g. RNA-transfected DCs should not be matured with polyI:C as this might hinder protein translation [25]. TNFα and IL-1ß are frequently used as the basic cytokine combination to induce pathway committed DCs, which are pro-inflammatory and migratory but still have capacity to mature further in vivo [12], e.g. by preparation of the injection site with proinflammatory substances such as imiquimod [11]. Our phenotypic data, showing that TLR-P DCs exhibit significantly higher maturation marker expression such as CD80 and CD83 than CDCs are in line with this and confirm previous reports that TLR-agonists induce full maturation in myeloid DCs [18, 26]. This fully mature phenotype was associated with a faster decline of DC numbers after prolonged in vitro culture, which can be explained with the higher amount of IL-10 produced by TLR-P DCs. Endogenous IL-10 production in TLR-matured myeloid DCs was demonstrated to inhibit accumulation of anti-apoptotic Bcl-2, which limits DC longevity [27]. Although the phenotype of DCs seemed to be rather stable over time, we cannot exclude a possible dedifferentiation of DCs e.g. into macrophages especially under the influence of IL-10. Notably, with the exception of MCP-1, all analyzed cytokine levels were higher after TLR-P maturation, which reached significance for IL-6, IL-8, IL-10, and IL-12_p35/40_, however, the IL-12_p35/40_/IL-10 ratio remained always positive. As expected, IL-12_p70_ production was greatly enhanced after TLR-P maturation [15, 28, 29], whereas in CDCs and iDCs, IL-12_p70_ remained below the detection limit. Although PGE_2_ is known to reduce IL-12_p70_ production in DCs via a cyclic adenosine monophosphate (cAMP-)dependent inhibition of the IL-12_p40_ subunit [30, 31], our TLR-P DCs still produced substantial amounts of IL-12_p70_, suggesting that the TLR-stimulus sufficiently compensates for the inhibitory PGE_2_ effect. Furthermore, considering migration times of 24 h from skin to the draining lymph node [32] and a relatively preserved IL12_p70_ production after 48 h, we postulate that TLR-P DCs injected after 16 h maturation will still be able to secrete active IL-12_p70_ at the DC:CD8^+^ interface in the lymph node.

In terms of de novo T-cell priming, CDCs and TLR-P DCs as well as the LPS/IFNγ DCs, which served as a positive control, were able to induce tumor-antigen-specific CD8^+^ T-cells at similar frequencies with a trend for higher proliferation of T-cells in the TLR-P group. The assay worked for tumor-associated peptides with an intermediate (Melan-A) and low (NLGNX and PTP) T-cell precursor frequency within the naïve T-cell repertoire, although the excellent MHC-binding affinity of the two latter glioma peptides (200–300 fold higher than for Melan-A_26-35L,_ according to the NetMHC4.0 algorithm) might also have contributed to optimal T-cell priming. Although the presence of IL-12 (which was secreted by our TLR-matured DCs) during priming can result in high avidity T cells [33], we could not observe differences in T-cell avidity after our priming assay with the caveat that this was an in vitro system only. An in vivo animal model has demonstrated that IL-12 deficient mice can mount protective CTL responses, when DCs were licensed by CD4 T-cell help, suggesting that DCs are able to use IL-12-independent pathways to prime naïve T cells [34]. Overall, methodologies which have demonstrated a benefit for T-cell function after contact with TLR-matured DCs varied substantially, ranging from increased allospecific proliferation of splenocytes [15, 35] to augmented IFNγ-production of TCR-transduced T-cells after cognate antigen contact [18], making a clear conclusion difficult. Watchmaker et al. reported that all DCs subtypes in their study were able to expand CD8^+^ T cells, but only type-1 polarized DCs induced Granzyme B and the peripheral tissue chemokines CCR7 and CCL5 on CD8^+^ T-cells. These findings are consistent with our data, showing similar frequencies of MHC-multimer^+^ CD8^+^ T cells but subtle differences in phenotype (effector T-cells) and integrin (VLA-4) expression. Therefore, we hypothesize that TLR-P DCs might confer the functional advantage of higher VLA-4 expression, which represents an important CNS-homing receptor, to primed CD8^+^ T-cells. Notably, in vivo administration of the TLR3-agonist, poly-ICLC also promoted VLA-expression on vaccine reactive CTLs [36].

A central finding of our study is that cross-presentation of protein antigens to CD8^+^ T-cell lines was diminished in TLR-P DCs, a process that was dose-dependent and required the presence of PGE_2_ during maturation. Ahmadi et al. had reported that in murine DCs the presence of PGE_2_ during differentiation and maturation resulted in a defective DC phenotype ultimately leading to abortive CD8^+^ activation. PGE_2_ is a member of a group of eicosanoids with pleiotropic effects on immune cells which are both context and dose-dependent [37]. In immune cells, PGE_2_ acts predominantly via the high-affinity EP_2_- and the low-affinity EP_4_-receptor [38]. In accordance with our data, triggering of the EP_4_-receptor at concentrations > 50 nM usually results in IL-12 and IL-23 reduction [39]. To the best of our knowledge, the biochemical pathways of PGE_2_ to interfere with antigen cross-presentation have not been described yet and require further mechanistic studies. It should be noted however, that we tested only soluble proteins for cross-presentation. Larger, particulate antigen, e.g. bigger fragments of tumor lysate might be handled differently, as uptake and allocation to intracellular compartments of antigens of distinct size and consistency follows different pathways [40].

In contrast to these latter findings, the pivotal role of PGE_2_ in augmenting DC migratory capabilities, mediated through activation of the CCR7 promotor via the p38/MAPK pathway, is well documented [7, 41, 42]. Although we and others have shown that PGE_2_ strongly enhances the CCR7-dependent in vitro migratory capacity in a dose-dependent way, in vivo this effect might be rapidly counteracted by CCR7-downregulation in CCL19-rich environments [43]. Indeed, imaging studies failed to demonstrate a benefit for accumulation of PGE_2_-matured DCs in secondary lymphoid organs [43, 44], making it uncertain whether this in vitro effect will translate into a clinical benefit. One limitation of our study is that all our readout assays were separate in vitro experiments. With these alone, the resulting net effect in vivo is difficult to predict. Therefore, further preclinical in vivo models are needed to assess the effects of PGE_2_ on migration, CTL priming, and cross-presentation of a DC-based cancer vaccine.

Sustained CD8^+^ effector T-cell responses, the aim of each cancer vaccine, require CD4^+^ T-cell help [45, 46], ideally from the same epitope, which can only be achieved when the antigen (synthetic long peptides or proteins) is processed intracellularly by DCs and the resulting peptide fragments are presented on both MHC class I and II molecules [47]. In contrast, for short peptides, loading on pre-activated DCs was the best way to induce protective antitumor CTL responses [48]. Therefore, as one possible conclusion from our study, for DCs intended to be loaded exogenously with short peptides the TLR-P combination might be the right choice for maturation as suggested by Boullard et al. and others [19, 25], since TLR-P DCs are fully mature, produce IL-12_p70_, successfully prime CD8^+^VLA-4^+^ T cells, and show superior migratory capabilities. More caution is warranted when the vaccine antigen requires intracellular processing before presentation. In these cases, CDCs might be the better choice, as cross-presenting functions are preserved and yet these DCs express costimulatory markers, exhibit targeted migration, and successfully prime naïve CD8^+^ T cells as well. Alternatively, PGE_2_ in nanomolar concentrations could improve migratory functions while leaving no significant impact on cross-presentation. Thus, our data highlight an important new aspect of how different agents during maturation can impact DC functionality.

## Electronic supplementary material

Below is the link to the electronic supplementary material.

Supplementary file1 (PDF 532 kb)

## Abbreviations

7-AAD: 7-Aminoactinomycin
BDCA-3: Blood-derived dendritic cell antigen 3
CCL: Chemokine ligand
CCR: Chemokine receptor
CDC: Conventional, cytokine matured DC (here, matured with TNFα, IL-1ß)
COX-2: Cyclooxygenase 2
CMV: Cytomegalo virus
DC: Dendritic cell
FSC: Forward scatter
GMP: Good manufacturing practice
MACS: Magnetic activated cell sorting
MFI: Mean fluorescence index
TLR-P-DC: Monocyte-derived DC, matured with TLR3 + 7/8 agonists and PGE2
MDSC: Myeloid-derived suppressor cells
NLGN4X: Neuroligin-4, X-linked
PGE2: Prostaglandin E2
SSC: Sideward scatter
TCR: T-cell receptor
TLR: Toll-like receptor
PTP: Tyrosine-protein phosphatase non-receptor
VLA-4: Very late antigen-4 (α4ß1 integrin
