# Supplementary material for: Prostaglandin E2 in a TLR3- and 7/8-agonist-based DC maturation cocktail generates mature, cytokine-producing, migratory DCs but impairs antigen cross-presentation to CD8+ T cells
Source: Cancer Immunol Immunother. 2020 Feb 25;69(6):1029–42. doi: 10.1007/s00262-019-02470-1 (PMC7223547; doi:10.1007/s00262-019-02470-1)
Supplement: Supplementary file 1 — Supplementary file1 (PDF 532 kb) [file 262_2019_2470_MOESM1_ESM.pdf]

Supplementary Figure 1

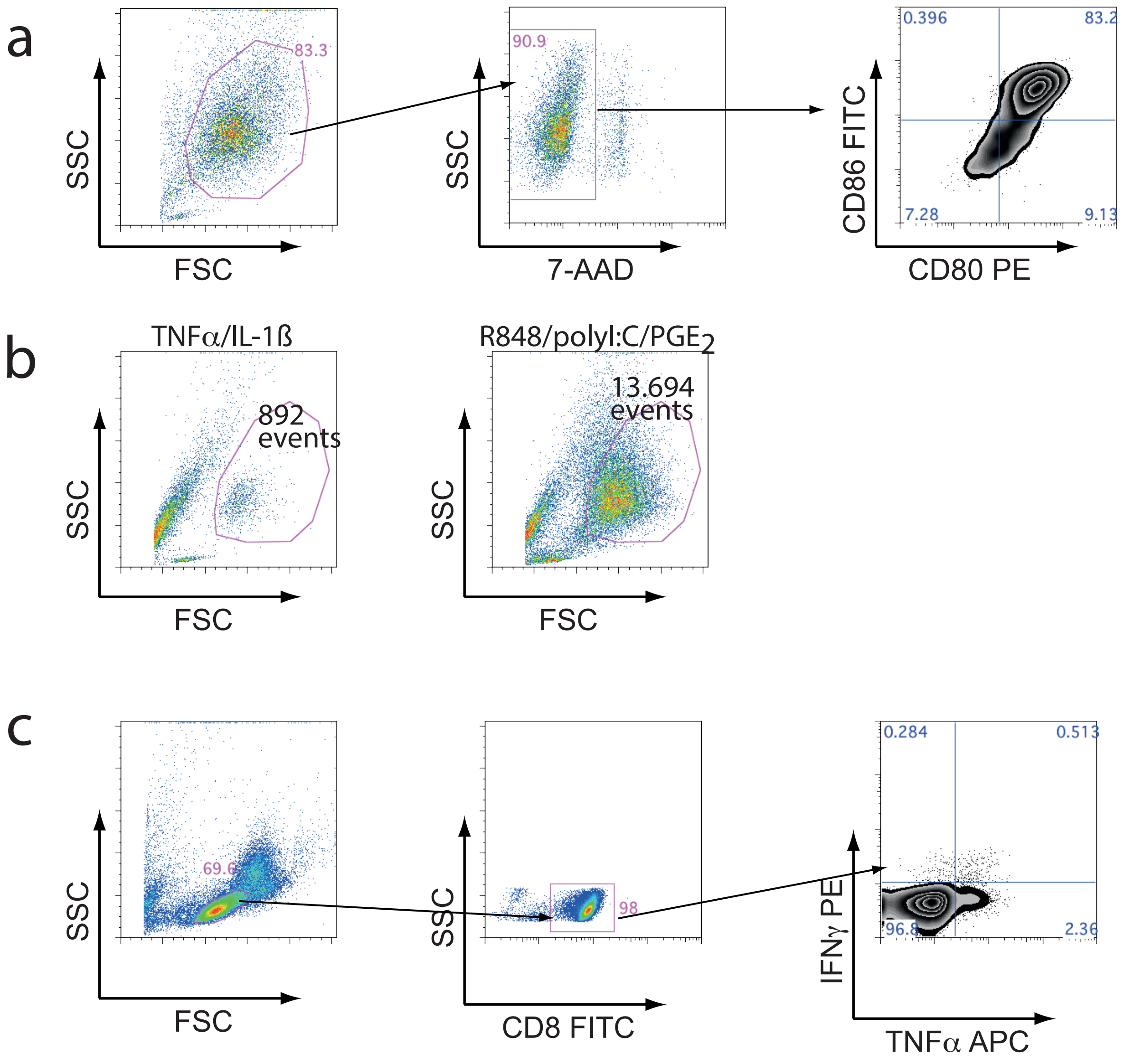

Supplementary Figure 1:

Gating strategies for different flow cytometry analyses. (a) Gating strategy for analyzing DC phenotypes in Fig. 1. After identifying DCs in a $SSC^{hi}FSC^{hi}$ gate, dead cells were removed by 7-AAD exclusion. $7\text{-}AAD^{neg}$ events were plotted for the respective marker combinations. (b) Gating strategy for counting and phenotyping migrated DCs from the lower chamber of transwell plates in a $SSC^{hi}FSC^{hi}$ gate for Fig. 2a-c. (c) Gating strategy for Fig. 3c: after identifying lymphocytes in $SSC^{low}FSC^{low}$ and gating on $SSC^{low}CD8^{+}$, cytokine producing cells were determined in zebra plots using quadrant statistics.
